# Versatile biocatalysis of fungal cytochrome P450 monooxygenases

**DOI:** 10.1186/s12934-016-0523-6

**Published:** 2016-07-18

**Authors:** Pradeepraj Durairaj, Jae-Seoun Hur, Hyungdon Yun

**Affiliations:** Korean Lichen Research Institute, Sunchon National University, Suncheon, South Korea; Department of Bioscience and Biotechnology, Konkuk University, Seoul, South Korea

**Keywords:** Cytochrome P450, Cytochrome P450 reductase, Monooxygenase reaction, Biocatalysis, Fungi

## Abstract

Cytochrome P450 (CYP) monooxygenases, the nature’s most versatile biological catalysts have unique ability to catalyse regio-, chemo-, and stereospecific oxidation of a wide range of substrates under mild reaction conditions, thereby addressing a significant challenge in chemocatalysis. Though CYP enzymes are ubiquitous in all biological kingdoms, the divergence of CYPs in fungal kingdom is manifold. The CYP enzymes play pivotal roles in various fungal metabolisms starting from housekeeping biochemical reactions, detoxification of chemicals, and adaptation to hostile surroundings. Considering the versatile catalytic potentials, fungal CYPs has gained wide range of attraction among researchers and various remarkable strategies have been accomplished to enhance their biocatalytic properties. Numerous fungal CYPs with multispecialty features have been identified and the number of characterized fungal CYPs is constantly increasing. Literature reveals ample reviews on mammalian, plant and bacterial CYPs, however, modest reports on fungal CYPs urges a comprehensive review highlighting their novel catalytic potentials and functional significances. In this review, we focus on the diversification and functional diversity of fungal CYPs and recapitulate their unique and versatile biocatalytic properties. As such, this review emphasizes the crucial issues of fungal CYP systems, and the factors influencing efficient biocatalysis.

## Background

Cytochrome P450 (CYP) monooxygenases, the ubiquitous enzymes with catalytic versatility, substrate diversity and atypical kinetics are one of the most fascinating targets for biocatalysis and play diverse roles in biotechnology, medicine and bioremediation [1–3]. The tetrapyrrole heme-thiolate CYP enzymes can catalyse the conversions of various hydrophobic as well as lipophilic compounds to more hydrophilic products in the presence of oxygen and cofactor NAD(P)H with or without an electron transfer system. Indeed, CYP enzymes are certainly the nature’s most versatile and promising catalysts [1, 4] owing to their varied and multifunctional characteristics: (a) CYPs are structurally diverse and functionally versatile enzymes that can perform rate-limiting and irreversible reactions in biosynthetic pathways, (b) CYPs catalyze the regiospecific and stereospecific oxidation of non-activated hydrocarbons, which is intricate to accomplish through chemocatalysis, and (c) CYPs are unique chemoselective and enantioselective enzymes involved in the production of high-value products [5–8]. Hitherto, various aspects of CYPs have been extensively reviewed, including its evolutionary paths, structure and function, complexity and diversity of CYP reactions, biological variations of electron transport chains, synthetic and catalytic applications, and the regulation and engineering of CYP systems [5–13].

CYP superfamily of enzymes exist in all biological domains and their presence predates the emergence of oxygen-metabolizing life forms [14]. Interestingly, the divergence of CYPs in fungal kingdom is enormous, and are involved in the synthesis of a wide range of primary and secondary metabolites, as well as in the degradation of environmental pollutants [15, 16]. Being a ubiquitous organism, fungi inhabitants diverse ecological niches, and adapts to various sources of carbon and nitrogen for their survival and metabolism [15]. Recent study based on high-throughput sequencing reveals that about 5.1 million fungal species exist on earth, which is about 6 times the total number of plant species [17]. Comprehensive biochemical analysis of molecular mechanisms showed that fungal adaptions are often facilitated by CYP monooxygenases [15]. Fungal CYPs play an essential role in their adaptations to ecological niches due to their diverse roles in the production of metabolites critical for pathogenesis, detoxification of xenobiotics and exploitation of substrates [15, 16]. In recent decades, fungal CYPs have emerged as a revolutionary system and have gained widespread attention for biocatalysis due to their fascinating and extraordinary metabolic diversity. While their distinguished role in primary and secondary metabolite synthesis fascinates biochemists and enzymologists, their xenobiotic detoxification and degradation properties captivates pharmacologists and toxicologists.

Although CYPs have been extensively reviewed [5–12], the limited number of reviews on fungal CYPs [15, 16, 18] calls for an updated article to highlight novel and crucial findings. We comprehensively summarized the outlooks of fungal CYPs in terms of divergence, classification, electron transport system, and their recent advancements. A compilation of novel fungal CYPs and their functional significances with respect to versatile biocatalytic potentials has been explicated. Furthermore, we elaborate on critical parameters involved in the heterologous expression and optimization of CYPs for efficient biocatalysis.

## Divergence of fungal CYPs

Evolution of CYPs corresponds to the organismal adaptation to diverse ecological niches, and the chemical warfare in synthesizing/neutralizing toxic metabolites among biological kingdoms [14]. The distinctive feature of fungi to survive on harsh environmental conditions, its extraordinary defense mechanic systems and their ability to produce a wide variety of primary and secondary metabolites has mainly contributed to the evolution and diversification of fungal CYPs. Apparently, the divergence of CYPs in the fungal kingdom is manifold compared to other biological kingdoms, leading to a tremendous diversification of CYPs to meet the metabolic needs [9, 15, 16, 18]. In general, the genome size of fungi is smaller than in plant and animal kingdoms ranging from 8.97 to 177.57 mb [19]. Although, the average genome size of ascomycota and basidiomycota species are 36.91 and 46.48 mb, respectively [19], the ratio of CYPs and open reading frames can vary from 0.04 % (*Saccharomyces cerevisiae*) to 2.06 % (*Postia placenta*) (Table 1). Fungal genome-sequencing projects have revealed the existence of >6000 fungal genes that code for putative CYPs, which yet have to be explored for novel catalytic enzymes [15, 16, 20]. To date, a vast number of CYPs have been identified in >2500 fungal species, and these are classified into ≈400 CYP families (namely CYP51-CYP69, CYP501-CYP699, and CYP5001-CYP6999) [21, 22]. Indeed, fungi comprise the largest number of CYP families/subfamilies, far outnumbering other kingdoms such as bacteria (333 CYP families), plants (127 CYP families), vertebrates (19 CYP families), and insects (67 CYP families), reflecting the enormous evolutionary and functional diversity of CYPs in the fungal kingdom [23]. Interestingly, certain fungal species such as *P. placenta* (353 CYPs) also possess the highest CYP counts (Table 1), as compared with species in other domains, namely *Mus musculus* (102 CYPs), *Anopheles gambiae* (105 CYPs), and *Streptomyces avermilitis* (33 CYPs), with the exception of plants (*Oryza sativa*, 455 CYPs) [2].

**Table 1:** Representative distribution of putative CYPs and CPRs across fungal phyla

Fungal Phyla		Species	Genome size (Mb)	ORF	No. of putative CYP	Ratio of CYP/ORF (%)	No. of putative CPR
Ascomycota	Pezizomycotina	*Magnaporthe oryzae*	45.0965	11,069	107	0.97	1
		*Neurospora crassa*	41.1024	9935	43	0.43	1
		*Aspergillus fumigatus*	29.385	9887	77	0.78	2
		*Aspergillus oryzae*	37.1178	12,063	163	1.35	2
		*Aspergillus niger*	34.8533	11,200	154	1.38	2
		*Aspergillus nidulans*	30.2427	10,568	120	1.14	2
		*Cochliobolus lunatus*	35.4974	NA	NA	NA	2
		*Fusarium graminearum*	36.6676	13,339	118	0.88	3
		*Fusarium oxysporum*	61.4707	17,735	169	0.95	4
		*Fusarium verticillioides*	41.8851	14,199	129	0.91	1
		*Penicillium chrysogenum*	32.5255	12,791	101	0.79	NA
	Saccharomycotina	*Saccharomyces cerevisiae*	14.2673	6692	3	0.04	1
		*Candida albicans*	27.5589	6090	10	0.16	1
		*Candida tropicalis*	15.3268	6258	12	0.19	1
		*Yarrowia lipolytica*	20.6238	6524	17	0.26	1
	Taphrinomycotina	*Schizosaccharomyces pombe*	12.5913	5058	2	0.04	1
Basidiomycota	Agaricomycotina	*Phanerochaete chrysosporium*	29.8426	10,048	145	1.44	1
		*Postia placenta*	90.8919	17,173	353	2.06	NA
	Pucciniomycotina	*Puccinia graminis*	88.7244	20,567	18	0.09	1
	Ustilaginomycotina	*Ustilago maydis*	19.6644	6689	22	0.33	NA
Zygomycota		*Rhizopus oryzae*	47.5346	17,482	49	0.28	2
		*Phycomyces blakesleeanus*	53.9	14,792	56	0.38	NA
Chytridiomycota		*Batrachochytrium dendrobatidis*	24.3151	8732	9	0.1	NA

The genomic information is obtained from the genome browser in NCBI (http://www.ncbi.nlm.nih.gov/genome/browse/). The data information on ORFs and putative CYPs were obtained from fungal cytochrome P450 database (http://p450.riceblast.snu.ac.kr/species.php) and Dr. Nelson’s cytochrome P450 database (http://drnelson.uthsc.edu/fungal.genomes.html), and information on putative CPRs was obtained from Ref. [78]

*ORF* open reading frame, *NA* information not available

Nevertheless, the extraordinary functional and evolutionary diversity of fungal CYPomes has complexified the classification of fungal CYPs. The classification of CYPs is generally based on their amino acid sequence similarity, i.e., sequences with >40 % similarity form a single CYP family and sequences with >55 % similarity form subfamilies [24]. Remarkably, despite their wide diversity and low sequence similarities, fungal CYPs possess four signature motifs, along with their preserved tertiary structures and enzymatic functions, that facilitate the identification of CYPs from the fungal genome: (i) FXXGXRXCXG, the heme-binding domain enclosing the invariant Cys ligand to the heme; (ii and iii) motifs EXXR and PER, which form the E-R-R triad retains the position of heme pocket and stabilizes the core structure; and (iv) AGXDTT, the oxygen-binding and activation domain [14, 25–27]. Nevertheless, despite the widely recognized conservativeness, the motifs are distinguishable among the taxonomic groups, and the most clear distinction was observed in prokaryotes, probably due to the early evolutionary divergence [22]. Recent investigations of the 47 completed fungal genomes suggest that the two large gene duplications and the horizontal gene transfer in Ascomycota and Basidiomycota contributes to the diversification of fungal CYP superfamily [22]. Apparently, despite their high divergence, fungal CYPs can be clustered into 15 clades based on their phylogenetic relationships [22]. This work provides clear insights into the evolutionary scenario of fungal CYP superfamily based on their phylogenetic and taxonomic relationships [28]. Interestingly, the highly conserved and consistent *CYP51* and *CYP61* global families act as molecular clocks and specifies the individualization of CYPomes in the fungal kingdom [21].

## CYP catalytic cycle and fungal CYP systems

**Fig. 1:**
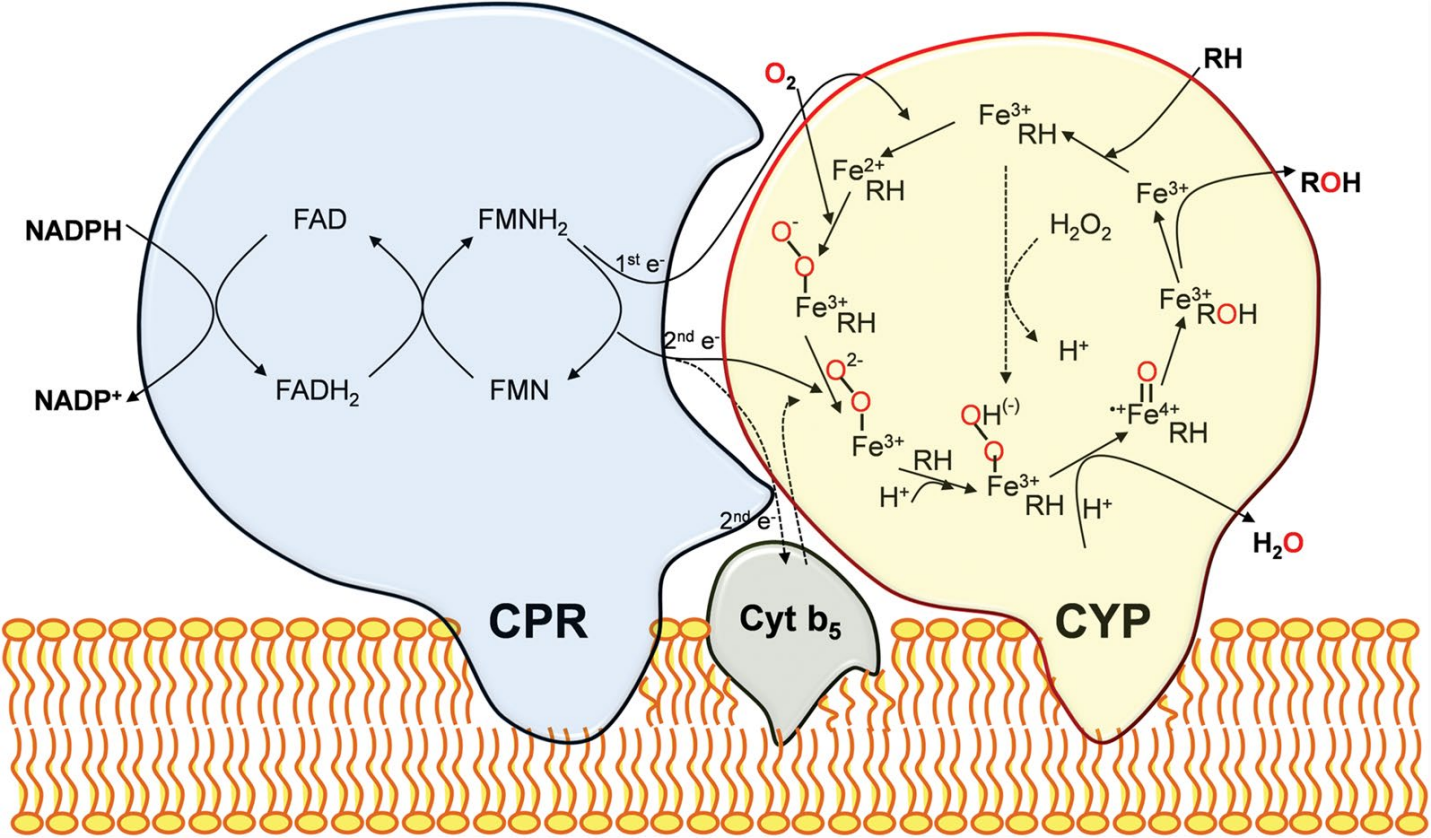
CYP catalytic cycle and schematic representation of the interaction of CYP-CPR for class II CYP system

CYPs catalyse the monooxygenase reaction (RH + O_2_ + 2e^−^ + 2H^+ ^→ ROH + H_2_O) by the insertion of one of the atoms of molecular oxygen into the substrate, while the second oxygen atom is reduced to water [9, 29]. CYP-catalyzed substrate hydroxylation follows a general catalytic mechanism (Fig. 1): (a) ‘substrate binding’ is the first process in the catalytic cycle, where the substrate (RH) enters the active site and binds to the heme iron of CYP, which is in the oxidized state (Fe^3+^); (b) the ‘first reduction step’ involves the transfer of one-electron to the ferric heme iron (Fe^3+^) by cytochrome P450 reductase (CPR), followed by the binding of molecular oxygen, thereby reducing it to form the ferrous dioxy complex (Fe^2+^–O_2_); (c) the ‘second reduction step’ involves the transfer of the second electron and a proton either from the CPR or from cytochrome b5 (Cyt b5) yielding a ferric hydroperoxy complex (Fe^3+^–OOH); (d) heterolytic cleavage of the O–O bond and the second protonation accompanied by the generation of a H_2_O molecule forms the reactive ferryl-oxo intermediate (Fe^4+^=O, porphyrin π-cation radical); (e) in the ‘product formation’ step, abstraction of a H_2_ atom followed by the radical recombination results in the formation of the hydroxylated product (ROH); and (f) the final step is dissociation of the oxidized substrate from the active site of CYP, wherein the enzyme returns to its initial ferric state (Fe^3+^) and is thus equipped to react again. An alternative route is binding of H_2_O_2_ to the ferric heme iron, which leads to the peroxide shunt pathway [5, 30].

**Fig. 2:**
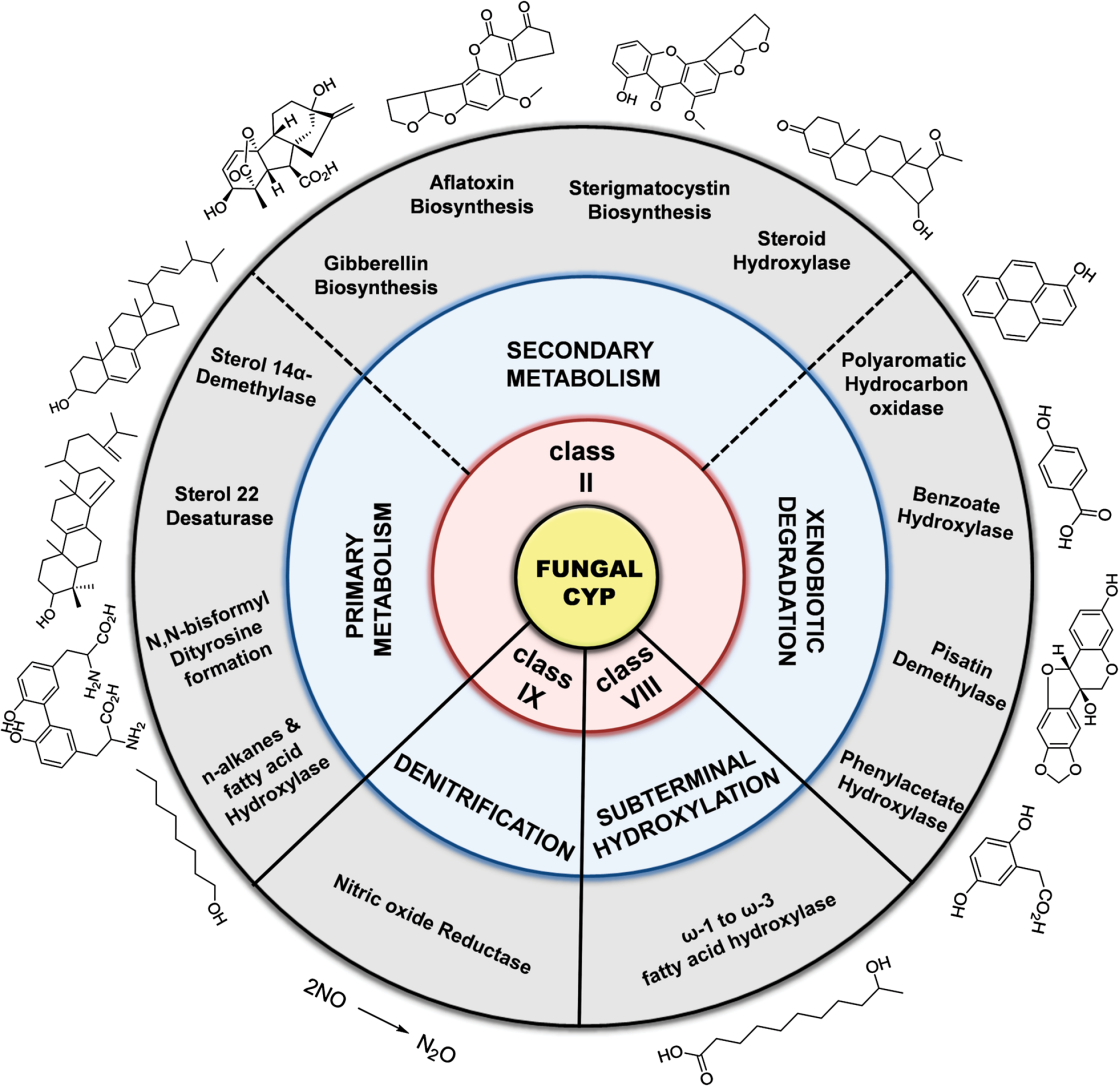
Representative scheme of functional diversification and classification of fungal CYP systems. Categorization of the functional properties of Class II CYP systems based on the primary metabolism, secondary metabolism and xenobiotic detoxification was perceived from Ref. [16]

With regard to the aforementioned catalytic process, CYPs employ highly diversified redox chains for the electron transfer mechanism. Depending on the topology of the protein components involved in electron transfer, CYP systems are classified into 10 different classes, and the fungal CYPs fall into classes II, VIII, and IX systems [13, 16]. Class II comprises two integral membrane proteins: CYP and CPR containing the prosthetic cofactors FAD and FMN, which deliver two electrons from NAD(P)H to the heme moiety (Fig. 1). Alternatively, it may also comprise a third protein component, Cyt b5, which transfers a second electron to the oxyferrous CYP. Rarely, certain CYPs (*CYP5150A2*) can be directly activated by Cyt b5 and NADH-dependant Cyt b5 reductase (CB5R) in the absence of CPR [31]. Most of the fungal CYPs belong to class II and perform extremely diverse catalytic reactions (Fig. 2). Interestingly, in the class VIII, fungi encompass fused proteins in which the N-terminal heme domain is fused with a C-terminal diflavin reductase partner (CPR) via a short protein linker (P450foxy) [32]. Herein, the electrons are transferred from NADPH to the active site of CYP through its reductase domain. Class VIII CYPs are catalytically self-sufficient and perform subterminal hydroxylation of fatty acids, closely resembling a bacterial CYP (P450BM3) (Fig. 2). Exceptionally, class IX is composed of single protein, whereby electrons are directly transferred from NAD(P)H to CYP without the need for any additional redox partners (P450nor) [32]. Class IX differs functionally from the rest of the CYPs because they catalyze the reduction of two molecules of NO to N_2_O. Interestingly, P450nor is the only soluble fungal CYP discovered so far, and performs denitrification (Fig. 2).

## Functional diversity of fungal CYPs

**Fig. 3:**
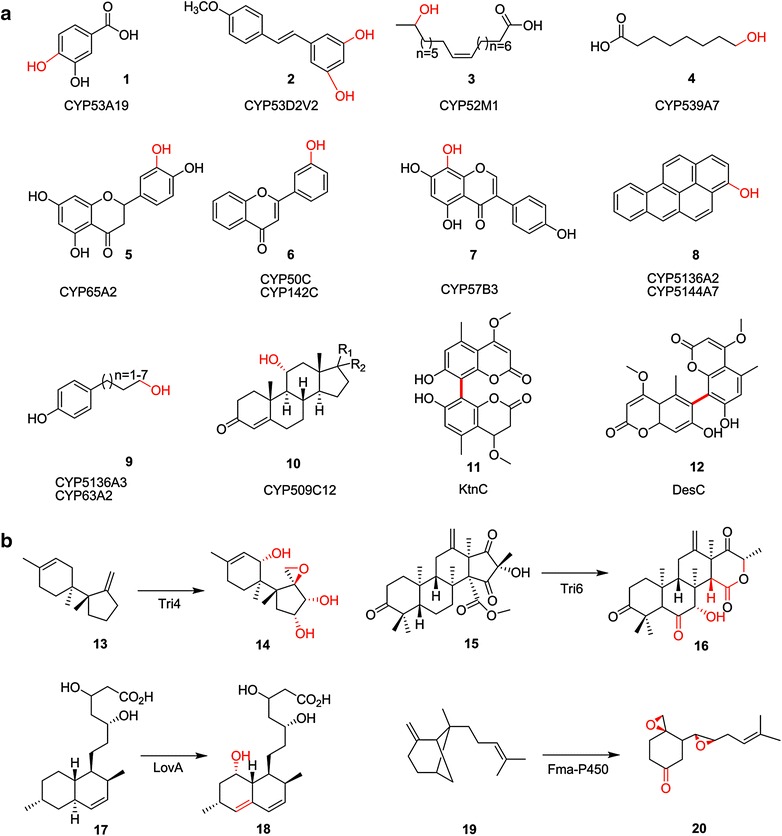
Versatile monooxygenase reactions catalysed by fungal cytochrome P450 enzymes. **a** Selected examples of reaction products of fungal CYPs. **b** Selected examples of multifunctional reactions of fungal CYPs. The products were generated through multiple consecutive catalytic reaction mediated by a single CYP. Newly introduced hydroxyl groups and bonds are shown in *red*

In the fungal kingdom, CYPs are involved in the biosynthesis of various primary and secondary metabolites with high substrate specificity. Some notable examples of the fungal primary metabolism are housekeeping functions such as ergosterol biosynthesis, meiotic spore-wall biogenesis, and *n*-alkane hydroxylation, whereas fungal secondary metabolism deals with the biosynthesis of hormones, mycotoxins, and the like (Fig. 2) [16]. Fungal CYPs are also capable of detoxifying and degrading various xenobiotic compounds encountered in their environments, such as polycyclic aromatic hydrocarbons *(*PAHs), phenolic compounds, and other toxic environmental pollutants [16]. There have been ample studies regarding fungal CYP-mediated reactions, and these reports have been reviewed [16]. In recent years, the discovery of several novel fungal CYPs has further expanded the scope of their biotechnological and industrial applications. Some of the exceptional and versatile reactions of fungal CYPs will be described in this review and are structurally illustrated (Fig. 3).

One of the well-studied fungal CYPs is the omnipotent *CYP51* enzyme responsible for oxidative removal of the 14α methyl group of eburicol and lanosterol to form ∆14,15-desaturated intermediates. Interestingly, in contrast to the general fungal *CYP51* enzymes, *MgCYP51* from *Mycosphaerella graminicola* demonstrated selectivity, sterol specificity, and temperature sensitivity by demethylating only eburicol, but not lanosterol at low temperatures (22 °C) [33]. Advances in this area have been crucial in the development of azole-based antifungal drugs targeted towards *CYP51* enzyme, which inhibits ergosterol biosynthesis and causes disruption of cell membrane [34, 35]. However, the development of resistance by fungal species, and the metabolic similarities within the fungal kingdom as well as higher eukaryotes constraints the effective treatment against fungal infections [36]. Strikingly, *CYP53* emerges as an ideal target for the development of more selective antifungal treatment, as this homolog is native only to pathogenic fungi and it plays crucial roles in both fungal primary (β-ketoadipate pathway) and secondary metabolism (detoxification of phenolic compounds) [36, 37]. Though the sole *CYP53* metabolism is through the benzoate-para-hydroxylation, the substrate specificity of *CYP53* enzymes may vary among fungal (ascomycetous and basidiomycetous) species [37–39]. Interestingly, *CYP53A19* of *Fusarium oxysporum* performs the hydroxylations of benzoic acid and 3-hydroxybenzoic acid (converted into **1**) (Fig. 3a), as well as the demethylation of 3-methoxybenzoic acid [38]. In contrary to the general hypothesis that *CYP53* exhibits enzyme-substrate binding only towards the carboxyl group of benzoate derivatives, it is worth mentioning that *CYP53* also shows substantial specificity towards methoxyl group (s) in the stilbene derivatives [40]. In *P. placenta*, *CYP53D2* performs the *O*-demethylation activity against 3,5,4′-trimethoxy-*trans*-stilbene and 3,5-dimethoxy-*trans*-stilbene to produce 3-hydroxy-5,4′-dimethoxy-*trans*-stilbene and 3,5-dihydroxy-4′-methoxy-*trans*-stilbene, respectively (**2**) [40].

Terminally oxidized omega hydroxy fatty acids (ω-OHFAs) are multifunctional compounds involved in the production of various industrial products with broad commercial and pharmaceutical implications [41]. However, due to the selectivity and controlled reactivity in C-H oxygenation reactions, the chemosynthesis of ω-OHFAs is intricate to accomplish. Interestingly, fungal CYPs are involved in the initial and rate limiting step of hydroxylation of *n*-alkanes and fatty acids [42, 43]. In *Starmerella bombicola*, members of the *CYP52* family (*CYP52M1*, *CYP52E3*, and *CYP52N1*) performed the ω- and ω-1 hydroxylation of various fatty acids (**3**) [44]. *CYP52M1* is involved in biosynthesis of sophorolipid, whereas *CYP52E3* and *CYP52N1* might be involved in the alkane metabolism [44]. Members of the *CYP52* family have also been identified in the entomopathogenic fungi *Metarhizium robertsii* (*MrCYP52*) and *Beauveria bassiana* (*CYP52X1*) and are primarily involved in alkane and insect epicuticle degradation [45, 46]. In *F. oxysporum*, *CYP539A7* catalyzes the regioselective hydroxylation at the ɷ-position of caprylic (**4**), capric, and lauric acid, whereas *CYP655C2* was reactive only towards capric and lauric acid [41]. Correspondingly, *CYP630B18* from *Grosmannia clavigera* performs the highly specific ω-hydroxylation towards oleic acid [47]. Fungal CYPs are also involved in the hydroxylation of flavonoids, a significant secondary metabolite with a wide range of potential pharmacological applications [48, 49]. In *P. chrysosporium,CYP65A2* catalyzes the 3′-hydroxylation of naringenin to yield eriodictyol (**5**) [48], whereas *CYP50C* (*CYP5147A1*) and *CYP142C* (*CYP5136A1*) catalyze the 3′-hydroxylation of flavone (**6**) and *O*-deethylation of 7-ethoxycoumarin [49]. In *A. oryzae, CYP57B3* converts the isoflavonoid genistein into 8-hydroxy- (**7**), 6-hydroxy-, and 3′-hydroxy-genistein [50].

Remarkably, certain fungal CYPs (*CYP5136A2*, *CYP5145A3*, *CYP5144A7*, *CYP5136A3*, *CYP5142A3*, and *CYP5144A5*) have an extraordinary capability to degrade and/or mineralize the recalcitrant PAHs [51]. In *P. chrysosporium,CYP63A2* and *CYP5136A3* oxidize structurally diverse hydrocarbons such as fused-ring high-molecular-weight PAHs (**8**), endocrine-disrupting long-chain alkylphenols (**9**), and crude oil aliphatic hydrocarbon *n*-alkanes [52, 53]. In addition, CYP members of *P. placenta* (*CYP5150*, *CYP5027,* and *CYP5350*) are also involved in the oxidation of a series of PAHs such as anthracene, carbazole, phenanthrene, and pyrene [40]. Interestingly, the chemically complex stereo- and regioselective hydroxylation steps involved in the production of steroid drugs are performed by certain filamentous fungi mediated by CYP enzymes [54]. In *Rhizopus oryzae,CYP509C12* performed steroid hydroxylation at the 11α and 6β positions towards various substrate spectra, including progesterone, testosterone, 11-deoxycorticosterone, and 11-deoxycortisol (**10**) [55]. Furthermore, members of *CYP512* along with *CYP5139* and *CYP5150* from *P. placenta* also showed substantial reactivity towards steroidal compounds, primarily testosterone [40]. Recently, CYPs capable of performing complex regioselective and stereoselective bimolecular phenoxy radical couplings have been identified in fungi. The bicoumarin synthase CYPs KtnC (from *Aspergillus niger*) and DesC (from *Emericella desertorum*) performed the regio- and stereoselective biaryl coupling of the same monomeric coumarin 7-demethylsiderin and formed the 8,8′-dimer *P*-orlandin (**11**) and *M*-desertorin A (**12**), respectively [56].

Fungal CYPs also appear as multifunctional biosynthetic enzymes performing consecutive multiple catalytic processes, which differ from their substrate promiscuity or broad substrate specificity (Fig. 3b). *F. fujikuroi* harbor four multifunctional CYPs (*CYP68A1,CYP68B1*, *CYP69A1*, and *CYP503A1*) and performs 10 of the 15 biosynthetic steps towards the production of gibberellins [57]. In *F. verticillioides, CYP505* (Fum6) is involved in the two consecutive hydroxylation reactions at carbons C-14 and C-15 for the synthesis of fumonisin polyketide [58]. Similarly, in *F. graminearum*, *CYP58* (Tri4) is involved in the four consecutive oxygenation steps from trichodiene (**13**) to isotrichotriol (**14**) (one epoxidation and three hydroxylations) towards trichothecene skeleton formation [59]. In *A. terreus* during the biosynthesis of terretonin, the CYP enzyme Tri6 catalyzes three successive oxidations to transform terrenoid (**15**) into an unstable intermediate (**16**) [60]. Similarly, during the lovastatin biosynthesis in *A. terreus*, LovA performed two central consecutive oxidations (introduction of 4α,5-double bond and C-8 hydroxylation), in which LovA catalyzed the conversion of dihydromonacolin L acid (**17**) to monacolin L acid and then to monacolin J acid (**18**) [61, 62]. Furthermore, in *A. fumigatus,* the biosynthetic gene cluster involved in the fumagillin biosynthetic pathway encompasses a multifunctional Fma-P450 (*Af*510), which performs successive hydroxylations, bicyclic ring-opening, and two epoxidations to convert β-trans-bergamotene (**19**) into 5-keto-demethoxyfumagillol (**20**) [63]. Interestingly, fungal kingdom encompasses an allied group of CYP like heme proteins, unspecific peroxygenases (UPO, EC1.11.2.1), performing peroxide-driven substrate oxidation [64]. It combines the catalytic cycle of heme peroxidases with the “peroxide shunt” pathway of CYPs, and prefers H_2_O_2_ over NAD(P)H. Some of the notable fungal peroxygenases are *Aae*UPO, *Cra*UPO and *Mro*UPO, and are involved in the oxidation of halides, aryl alcohols, naphthalene, bromide as well as bulkier substrates [64]. Furthermore, fungi also possess self-sufficient and functionally linked DOX-CYP fusion enzymes. Herein, the N-terminal dioxygenase (DOX) domains are homologs to animal heme peroxidases, and their C-terminal CYP domains functions on peroxide shunt pathway [65]. The fungal DOX-CYP gene family has five subfamilies and recently a new member of 10*R*-DOX-EAS (MGG_10859) capable of performing epoxy alcohol synthase activities was identified in *Magnaporthe oryzae* [65]. Although these group of enzymes are slightly different from classic CYP enzymes, it is worth mentioning their functional properties for further comprehensive analysis.

## Systematic approach to heterologous expression of fungal CYPs

In order to explore the enzymatic, structural and functional characteristics of fungal CYPs, the heterologous expression of enzymes is often required. Microbial cells serves as an excellent factories for heterologous enzyme production, representing about 90 % of the total biotransformation market [66]. Crucial for achieving their functional expression is the identification of an appropriate host, since handling fungal CYPs can be rather tricky because of their membrane-bound nature, low expression levels, protein instability, effective substrate uptake and tolerance and need for rich electron transfer cofactors [7]. In addition, the primary challenge in amplifying full-length cDNA is to determine appropriate mRNA profiles due to the existence of introns in the fungal DNA. In general, the expression of fungal CYP genes involved in secondary metabolite synthesis is rather complex and are mainly influenced by culture conditions [67]. Indeed, some of the fungal CYP-encoding genes are independently regulated and differentially expressed; several CYPs may be induced upon different xenobiotic aromatic and aliphatic compounds at the transcriptional level [51]. In *P. chrysosporium*, six PAH-responsive CYP genes identified by genome-wide microarray screening were induced and upregulated upon varying ring-sized PAHs, and their catalytic function towards various PAHs was determined using recombinant enzymes [51]. Furthermore, the induction of CYPs by pentachlorophenol (PCP) was also observed in *P. chrysosporium*, in which only the PCP-induced fungal microsomes led to the oxidation of PCP to form tetrachlorohydroquinone [68]. Remarkably, the transcriptional regulation of CYP-dependent metabolic pathways can be alternatively induced or upregulated by defined high or low nitrogen conditions, as observed in *P. chrysosporium, C. versicolor, P. placenta*, and *A. oryzae* [40, 69, 70]. Moreover, as fungal CYPs follow a time-dependent course of gene expression, the use of RNA cocktail mixtures isolated from different culture intervals (5–20 days) has proved to be successful for obtaining cDNA [38, 41, 69, 70]. The following sections will elaborate on the various microbial cell factories towards efficient functional expression and potential biocatalysis of fungal CYPs.

### Expression of fungal CYPs in bacterial host systems

*Escherichia coli is* the preferred bacterial host for heterologous protein expression due to its various advantages such as high growth rate, cost-effective culture media, potential for high cell density, ease in genetic manipulation and extensive knowledge on its genetics and physiology [71]. Besides, unlike other bacteria (e.g., *Streptomyces*), *E. coli* does not possess any native CYP that could interfere with the measurement of overexpressed CYPs. However, since most heterologous CYPs are expressed as apoproteins in *E. coli*, a heme precursor, 5-aminolevulinic acid, is supplemented during the induction of CYP or glutamyl-tRNA reductase, a key enzyme catalysing the rate-limiting reaction in heme biosynthesis, is often co-expressed [72]. Generally, *E. coli* is the ideal host for soluble bacterial CYPs because it permits substantial protein expression and purification. However, the expression of membrane-bound eukaryotic fungal CYPs often suffers in bacterial systems as a result of non-expression, protein misfolding, or aggregation into insoluble inclusion bodies, as well as slow substrate uptake and lack of compatible reductase systems [37, 38, 73]. For instance, bacterial expression of *CYP52A21* from *Candida albicans* resulted in no detectable CYPs in the soluble fraction [74]. Nevertheless, an anionic surfactant, CHAPS, facilitated the solubilization of active CYP protein from the membrane fraction with 60 nmol/L of expression (Table 2) [74]. Several approaches to overexpress membrane bound CYPs in *E. coli* already were proved to be efficient, such as N-terminal modifications, chaperonin co-expression, and artificial gene synthesis with optimized codon frequency [73]. However, most of the studies were carried out with mammalian CYPs, and only a few studies focused on fungal CYPs. Although, the truncation/modification of N-terminal transmembrane domain (TMD) resolves the problems associated with hydrophobicity, the approach turned out to be case-sensitive only improving expression of limited number of fungal CYPs [75]. One such example was recently observed for *CYP53A19* of *F. oxysporum*, where the truncation of the TMD region did not facilitate soluble protein expression and resulted in inactive CYP form [38].

**Table 2:** Representative examples of Fungal CYP expression and their kinetic parameters

CYP	CYP source	Expression host	Expression level	Substrate	CPR source	Kinetic parameters	Notes	Ref
*CYP53A1*	*A. niger*	*A. niger*	160 pmol/mg of protein (Microsomal fraction)	Benzoic acid	*A. niger*	*k*_*cat*_; 270 ± 30 nmol min^−1^ nmol P450^−1^, *K*_m_; 0.083 ± 0.009 mM	Para-hydroxylation	[94]
*CYP65A2*	*P. chrysosporium*	*S. cerevisiae*	1 nmol/L	Naringenin	*S. cerevisiae*	*k*_*cat*_; 0.29 ± 0.02 min^−1^, *K*_m_; 391 ± 27 μM	3′-Hydroxylation	[48]
*CYP50C*	*P. chrysosporium*	*S. cerevisiae*	40 pmol/mg of protein (Microsomal fraction)	Flavone	*S. cerevisiae*	*k*_*cat*_; 0.52 ± 0.03 min^−1^, *n*; 2.2 ± 0.1, *K*_S_; 109 ± 6 µM	3′-Hydroxylation, Hill equation was used for kinetic parameters	[49]
*CYP142C*	*P. chrysosporium*	*S. cerevisiae*	119 pmol/mg of protein (Microsomal fraction)	Flavone	*S. cerevisiae*	*k*_*cat*_; 0.019 ± 0.002 min^−1^, *n*; 2.0 ± 0.4, *K*_S_; 152 ± 30 µM	3′-Hydroxylation, Hill equation was used for kinetic parameters	[49]
*CYP52M1*	*S. bombicola*	*S. cerevisiae*	NA	Oleic acid	*Arabidopsis thaliana*	*k*_*cat*_; 535 ± 60 pmol min^−1^ mg P450^−1^, *K*_m_; 40 ± 2 µM	ω-Hydroxylation	[44]
*PcCYP1f*	*P. chrysosporium*	*P. pastoris*	NA	Benzoic acid	*P. chrysosporium*	*k*_*cat*_; 0.013 μmol min^−1^μmol P450^−1^, *K*_m_; 185 µM	Para-hydroxylation	[39]
*CYP5150A2*	*P. chrysosporium*	*E. coli*	200–300 nmol/L	4-Propylbenzoic acid	*P. chrysosporium*	Initial velocity (*μ*mol/min/*μ*mol of P450); 0.3 ± 0.1 with CPR, 0.6 ± 0.1 with CPR, Cyt b5 and 10.6 ± 0.3 with CB5R, Cyt b5	Formation of 4-(2-hydroxypropyl)benzoic acid, ∆ 13 form of *CYP5150A2* coexpressed with GroEL/ES. CB5R/Cyt b5 act as electron partner in the absence of CPR	[31]
*MgCYP51*	*M. graminicola*	*E. coli*	NA	Eburicol	*M. graminicola*	*k*_*cat*_; 0.13 min^−1^, *K*_m_; 33 µM	14α- demethylation, Sterol selective and Temperature sensitive enzyme	[33]
*CYP53A15*	*C. lunatus*	*E. coli*	800–900 nmol/L	Benzoic acid	Mammal	*k*_*cat*_; 1.4 ± 0.5 min^−1^, *K*_m_; 1.4 ± 0.2 mM	para-hydroxylation	[37]
*CYP52A21*	*C. albicans*	*E. coli*	60 nmol/L	Dodecanoic acid	Rat	*k*_*cat*_; 33 ± 1 min^−1^, *K*_m_; 57 ± 2 µM	ω-Hydroxylation	[74]
*CYP630B18*	*G. clavigera*	*E. coli*	3.1 μmol/L	Oleic acid	*G. clavigera*	NA	ω-Hydroxylation	[47]
304 CYP isoforms	*P. chrysosporium* and *P. placenta*	*E. coli*	43–1255 nmol/L	NA	–	NA	27 CYPs were expressed in active form with/without NTD modifications among 304 CYPs	[75]

*NA* information not available

Extensive heterologous expression of fungal CYP isoforms from *P. chrysosporium* and *P. placenta* were attempted using an *E. coli* expression system [75]. Among the 304 CYP isoforms studied, 27 CYPs were expressed in active form with/without deletion of the N-terminal hydrophobic domain, with the expression levels of some CYPs over 1000 nmol/L (Table 2) [75]. Currently, several approaches exist to improve the bacterial expression of fungal CYPs, and one such promising approach is the construction of chimeric CYPs [76]. To this end, the deletion and/or replacement of the hydrophobic N-terminal domains (NTD) acts as a membrane anchor to facilitate the bacterial expression of fungal CYPs [75, 76]. A large-scale screening identified 64 candidates including *CYP5348N1, CYP5348T3P, CYP5144C1, CYP5144C8,* and *CYP5348L1v2,* whose NTDs potentially increases the expression level of various CYPs in *E. coli* [76]. Especially, replacement of N-terminal amino acid sequences (M-S-L–L-L-A-A-T-L-F-L–H-S-R-Q-K-R-Y-P-L-) from *CYP5144C1* of *P. chrysosporium* promotes high-level expression for several fungal CYPs in bacterial system [75, 76]. For example, a chimeric *CYP5037E1v1* of *P. placenta* modified with the N-terminal sequence of *CYP5144C1* enabled 2330 nmol P450/L of expression, about 10 times higher than the non-chimeric sequence [75, 76]. Additionally, codon optimization for proline residues in the proline-rich region (P–P–G–P) may also facilitate enhanced heterologous expression [76]. Furthermore, factors such as reduction of secondary mRNA structures, bacterial codon usage, and vector and host strain selection, along with optimal culture conditions may also govern the bacterial expression of eukaryotic CYPs [73, 75, 76]. Despite various attempts to improve the overexpression of membrane-bound fungal CYPs in bacterial system, there is still no concrete theoretical and systematic approach, but improvements can be achieved only via trial-and-error process [76].

### Expression of fungal CYPs in yeast host systems

The expression of fungal CYPs in yeast systems has gained widespread attention owing to the rich endoplasmic reticulum combined with the higher eukaryotic protein machinery [38, 41, 77, 78]. The conventional yeast *S. cerevisiae* is the most preferred microbial cell factory for extensive enzyme production as well as for synthesis of value added chemicals in industrial scales [79, 80]. *S. cerevisiae* serves as an ideal host system for eukaryotic CYP expression as it possesses three well-characterized CYPs, and enables the expression of membrane-bound CYP genes without any genetic modifications/truncations [38, 50, 77]. In addition, it offers conventional metabolic engineering approaches such as increasing the precursor supply by varying pathway enzyme expression levels or knocking out competing pathways to enhance the CYP mediated biocatalysis [41]. Functional studies of heterologously expressed CYPs using *S. cerevisiae* can be performed by three different systems i.e., biotransformation, resting cell and in vitro (microsome) systems [44]. Though in vitro studies are precise, the purification and enzyme reaction with microsomes is rather intricate and sophisticated due to the technical difficulties associated with the isolation of active microsomal protein [15] Apparently, whole-cell-biotransformations of recombinant yeasts are often more feasible owing to its simplicity, enzyme stability, and efficient biotransformation [77, 81].

**Fig. 4:**
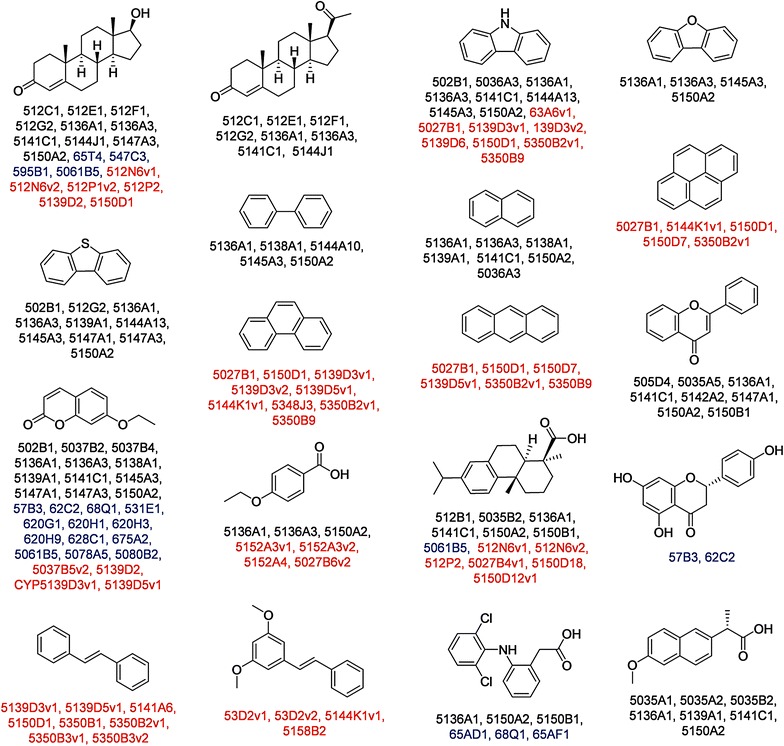
Catalytic potentials of fungal CYPs identified through functional screening. Representative examples of CYPs from *P. chrsosporium*, *A. oryzae* and *P. placenta* are presented in *black*, *blue* and *red*, respectively. The CYP names are denoted excluding “CYP”. For example, CYP512C1 is abbreviated as 512C1

Construction of cDNA libraries in *S. cerevisiae* is a promising strategy to develop a rapid functional screening system of multiple fungal CYPs. Functionomic studies carried out with 425 CYP isoforms from *P. chrysosporium* (120 CYPs), *P. placenta* (184 CYPs), and *A. oryzae* (121 CYPs) using *S. cerevisiae* CPR resulted in the discovery of several CYPs with novel catalytic potentials (Fig. 4) [18, 82]. Since the functional activity of the majority of fungal CYPs remains unclear and information regarding sequence-function relationships is limited, this specific screening system is certainly a competent approach to identify potential CYPs. Comprehensive functional screening of the *P. chrysosporium* CYP library facilitated the identification of a series of CYPs reactive towards seven petrochemicals, three plant-related compounds, three pharmacochemicals, three chlorinated dibenzo-*p*-dioxins, and two steroidal compounds [83]. Using the *P. placenta* CYP library, the active CYPs reactive towards 11 compounds, such as dehydroabietic acid, pyrene, and *trans*-stilbene were identified [40]. Furthermore, active CYPs from *A. oryzae* reactive towards 7-ethoxycoumarin, genistein, naringenin, testosterone, dehydroabietic acid, and diclofenac were also identified [50]. Construction and compilation of cDNA libraries is thus an efficient and effective strategy that allows rapid functional screening with ease in culturing several yeast transformants and prompt bioconversions. Nevertheless, construction of a full-length CYP library of a whole fungal CYPome is complex and laborious; this robust functional screening approach has proved successful and facilitated identification of several novel CYPs without prior information about their sequence-structure–function relationships.

However, there are some exceptions and all the cloned fungal CYPs may not necessarily express well in *S. cerevisiae*. For instance, among 120 CYP genes from *P. chrsosporium*, only 70 CYPs were successfully expressed based on a carbon monoxide binding assay [83]. Alternatively, unconventional non-*Saccharomyces* yeasts such as methylotrophic yeast *Pichia pastoris* [84], fission yeast *Schizosaccharomyces pombe* [85], dimorphic yeasts *Yarrowia lipolytica* [86] and *Arxula adeninivorans* [87], lactose-utilizing yeast *Kluyveromyces lactis* [88] and its thermophilic sister strain *K. marxianus* [88] can be employed to overcome the restrictions associated with heterologous CYP expression. For example, the expression of *P. chrysosporium* redox enzyme systems and CYPs (*CYP5136A3, CYP63A2*) in *P. pastoris* enhanced active form of functional expression [53, 89]. Interestingly, comparative heterologous expression and biotransformation studies of *CYP505A1*, a self-sufficient fungal CYP, was examined using a broad-range common vector (pKM118) system; *A. adeninivorans*, *Y. lipolytica*, and *K. marxianus* exhibited activity (in order from highest to lowest), whereas *S. cerevisiae* showed no *CYP505A1* activity [87]. These results suggest that the expression host should be carefully evaluated to permit the efficient functional characterization of membrane-bound fungal CYPs.

### Expression of fungal CYPs in fungal host systems

Biotransformation using fungal whole cells is a simple, low-cost, and time-saving system, thereby enabling direct and industrial-grade biocatalysis [90]. Several fungal conversions such as the hydroxylation of progesterone into 11α-hydroxyprogesterone in *Rhizopus* spp. and the 11β-hydroxylation of deoxycortisol in *Curvularia* spp. have indicated the commercial potential of fungal whole cell-based biocatalysis [1, 54]. Interestingly, filamentous fungi have emerged as a desirable expression host, especially for the large-scale production of pharmaceutically relevant homologous and heterologous proteins [91]. Although the manipulation of DNA in fungi is relatively complex, the fungal host systems offer proper maturation of mRNA precursors (splicing), which is rather difficult for other recombinant hosts (bacteria or yeast). In addition, fungal host system offers a platform for the eukaryotic-style post-translational modification of proteins because of its efficient protein secretion machinery [91–93]. However, as the secretory yields of heterologous proteins are comparatively lower than homologous proteins, various strategies were attempted to improve the recombinant protein production [92]. Interestingly, codon optimization prevents the premature polyadenylation and stabilizes the gene transcripts of heterologous genes for enhanced expression in fungal host system [92]. The membrane-bound benzoate para hydroxylase, *CYP53A1* and its redox partner, NADPH reductase from the filamentous fungus *Aspergillus niger* was overexpressed in the recombinant *A. niger* strain and functionally characterized using fungal microsomes (Table 2) [94]. In the fungal quadruple auxotrophic host *Aspergillus oryzae*, the whole gene cluster, including geranylgeranyl diphosphate synthase, terpene synthase, and two CYPs (PbP450-1 and PbP450-2) was introduced for the biosynthesis of diterpene aphidicolin [95]. Likewise, controlled expression of tenellin biosynthetic gene cluster including two fungal CYPs (*tenA* and *tenB*) from *Beauveria bassiana* was performed in the heterologous host *A. oryzae* [96]. Herein, replacing the promotors with heterologous *amyB* inducible promoter not only solved the transcriptional activation issues, but also significantly enhanced the productivity of tenellin (243 mg/L) over five times compared to its native host (47.3 mg/L) [96]. Alternatively, genetic engineering or manipulations of fungal gene clusters may facilitate the production of new secondary metabolite products. Expression of the hybrid polyketide synthetases in *A. oryzae* generated by rational domain swaps coupled with co-expression of CYP (*tenA* and *tenB*) genes facilitated resurrection of the extinct metabolite bassianin [97].

## Significance of CPR in fungal CYP-mediated reactions

Cytochrome P450 reductase, the membrane-bound diflavin electron donor protein, is crucial for CYP mediated reactions as it is responsible for the sequential delivery of two electrons for the activation of molecular oxygen in the class II system [29]. CPR has evolved as a fusion of two ancestral proteins in which the N-terminal domain is homologous with the bacterial flavodoxins containing FMN, while the C-terminal region is homologous with the ferredoxin NADP^+^ reductase and the NADH-*Cyt* b5 reductase containing FAD [16, 29]. In addition to the abundance of CYP, the efficacy of monooxygenase reactions also relies on the abundance and electron transfer compatibility of its redox protein partner [77, 98]. Apparently, compared to their larger and diverse CYPomes, most fungi possess only one or two CPRs, with an exception of four putative CPRs in *F. oxysporum* [Table 1] [38, 67, 78]. Although, the biological roles of multiple CPR paralogs in fungi remains unclear; it is evident that the functional role of each CPR is different [47, 67, 78]. It has been hypothesized that that CPR1 is responsible for CYPs during endogenous primary metabolism; while CPR2 possibly functions in secondary metabolism (xenobiotic detoxification) [47, 67]. The plant pathogenic fungus *Cochliobolus lunatus* possesses two reductases, CPR1 and CPR2; both supported CYP activity, whilst with different product specificities [67]. In the presence of CPR1, *CYP53A15* converted benzoic acid (BA) to 4-hydroxybenzoic acid and 3-methoxybenzoic acid (3-MBA) to 3-hydroxybenzoic acid (3-HBA). However with CPR2, the same enzyme converted both of the substrates to 3,4-dihydroxybenzoic acid through two step oxidations [67]. Likewise in the fungus *G. clavigera,* both the CPRs reduced cytochrome C and performed hydroxylation of oleic acid with *CYP630B18*; but the catalytic efficiency is much higher with CPR2 than with CPR1 [47].

Although fungal CYPs can show activity with heterologous CPR [40, 50, 83]; recent reports elucidated that the electron transfer compatibility and coupling efficiency of homologous CYP-CPR interactions are relatively higher [38, 77, 99]. The catalytic efficiency and substrate specificity of fungal CYPs was significantly influenced and altered by the source of reductase [38]. For instance, *CYP53A19* of *F. oxysporum* was reactive towards BA and 3-MBA in the presence of *S. cerevisiae* CPR; whereas it only converted BA with *C. albicans* CPR [38]. Remarkably, *CYP53A19* with its homologous *F. oxysporum* CPR not only increased the conversion rates of BA and 3-MBA, but also exhibited activity towards 3-HBA [38]. Similarly, *F. oxysporum* ɷ-hydroxylase CYPs, *CYP539A7* and *CYP655C2,* produced higher yields with its homologous redox partner compared to the heterologous partner [41]. As the interactions with CPR also play a major role in the outcome of CYP reactions, selection of an appropriate functional CPR is crucial to achieve optimal CYP activity. However, the underlying mechanism behind how CPRs affects substrate specificity beyond catalytic efficiency remains unclear. Hitherto, CPRs are perceived as highly conserved redox systems with not much room for functional or organismal diversity. Henceforth, much more attention, should be focused on CPR to enhance the catalytic efficiency and even to alter the substrate specificity of CYP-mediated reactions.

## Overcoming limitations and future perspectives

Although fungal CYPs have extensive potentials they suffer from certain limitations/challenges obstructing their viable applications. The general bottlenecks in industrial applications of CYPs are lack of stability, low activity, poor expression levels, limited solvent tolerance, expensive cofactor requirements, electron supply, and uncoupling between NAD(P)H oxidation and product formation [1, 100]. Considering the extraordinary potential of CYPs, several reviews have addressed their limitations and have focused on tackling these challenges to promote CYPs as robust biocatalysts [1, 3, 9, 10, 100]. Fungal CYPs also inherently possess the same limitations; and recent advancements has sought to overcome the major hurdles posed by fungal CYPs [1, 12]. Development of modern tools of biotechnology offers a wide scope in the protein discovery, structure prediction and enzyme engineering for the improvement of biocatalysts and their tailor-designed integration into the industrial processes. Enzyme engineering through mutagenesis is one of the tools to modify fungal CYPs as sustainable catalysts by solving issues concerning low expression levels and poor activity [1, 3, 10, 100]. For example, the oxidizing activity of the *P. chrysosporium* PAH oxidase (*CYP5136A3*) was significantly improved by a rational designing approach through site-directed mutagenesis [101]. In *A. terreus,* the expression levels of non-optimized LovA enzyme was very low and not detected in the immunoblot analysis using FLAG antibodies [61]. However, engineering of LovA through synthetic codon optimization and/or N-terminal peptide replacement from lettuce CYP markedly increased the expression levels in *S. cerevisiae* [61]. Interestingly, thermostable fungal CYPs from the thermophilic biomass-degrading fungi *Myceliophthora thermophila* and *Thielavia terrestris* identified using bioinformatic tools showed high thermal tolerance and in vitro stability based on calculated protein melting temperature and instability index [102]. Although further experimental characterization is necessary, such thermostable fungal CYPs can be used for industrial applications, and structural analysis may pave a way to enhance the stability of mesophilic CYP enzymes.

Remarkable achievements and strategies have been developed for several mammalian and plant CYPs to overcome these limitations [1, 6, 12]. The same approaches can also be applied to fungal CYPs to improve their functional properties. As the catalytic activity of CYPs is dependent on NAD(P)H and reductase, constant supply of electrons is crucial to enhance the efficiency of monooxygenases reactions. To surmount the electron dependence of fungal CYPs, construction of chimeric CYP-CPR fusion constructs or optimization of the redox chain can be effective. Artificial CYP-CPR fusion constructs termed “Molecular lego” are a competent startegy for comparative analysis of differential redox partners to find the optimal redox system [103, 104]. In addition, improved intracellular electron recycling can be obtained through cofactor regeneration by coexpressing CYPs along with glucose/formate dehydrogenase or aldehyde reductase [105]. Besides, alternative approaches such as surrogate oxygen atom donors, as well as enzymatic, direct chemical, electrochemical, photochemical and light driven cofactor-free reduction system, can also be employed [103, 106, 107]. Interestingly, immobilization of CYPs and their redox partners on nanodiscs is also a successful attempt in overcoming limitations in the assembly of CYP components [108]. Incorporation of membrane bound CYPs in nanodiscs enables stable, soluble, homogenous and monomeric preparations in native-like lipid bilayer environments thereby preventing aggregation and inactivation [108]. Furthermore, metabolic engineering of expression hosts are performed by knocking out competing pathways to enhance the yield of CYP-mediated biocatalysis. Engineering of the *C. tropicalis* genome by eliminating 16 undesirable genes comprising 6 CYPs, 4 fatty alcohol oxidases, and 6 alcohol dehydrogenases enabled effective production of ω-hydroxy fatty acids [43]. Likewise, construction of β-oxidation pathway inactivated (ΔPox1) *S. cerevisiae* mutant by disrupting the acyl-CoA oxidase enzyme has also proven successful for fungal CYP mediated biotransformations [41]. Remarkably, CYPs also play key roles in synthetic biology for the production of highly valuable compounds; development of the antimalarial drug artemisinin by engineering *CYP71AV1* from *Artemisia annua* in yeast was certainly a ground-breaking achievement [6, 109]. Although plant, mammalian, and bacterial CYPs are currently focused in synthetic biological studies, fungal CYPs will soon progress as a cynosure due to their outstanding functional diversity.

## Conclusion

This review summarizes the recent developments in the study of fungal CYP systems and elaborates on their novel catalytic properties. Realization of the versatile biocatalytic potentials and applications of fungal CYPs has led to the discovery of many novel enzymes. With the plethora of functional diversity and magnificent catalytic potentials, fungal CYPs have emerged as versatile biocatalytic enzymes. Nevertheless, a multitude of fungal CYPs remain unexplored and unexploited. Overcoming current challenges with the advent of modern biology and a better understanding of genome-structure-functionomic analysis will certainly open the window for various significant applications.

## Abbreviations

CYP: cytochrome P450
CPR: cytochrome P450 reductase
*Cyt b5*: cytochrome b5
